# Supplementary material for: Unraveling Connective Tissue Growth Factor as a Therapeutic Target and Assessing Kahweol as a Potential Drug Candidate in Triple-Negative Breast Cancer Treatment
Source: Int J Mol Sci. 2023 Nov 14;24(22):16307. doi: 10.3390/ijms242216307 (PMC10671558; doi:10.3390/ijms242216307)
Supplement: Supplementary file 1 [file ijms-24-16307-s001.zip › ijms-2589670-supplementary.pdf]

SI Table S1. Characteristics of TNBC patients with CTGF expression level

	CTGF_High (N=65)	CTGF_Low (N=32)	p-value
**Age**	53.3 ± 13.0	56.3 ± 11.3	0.271
**Status**			0.077
- 0:Living	53 (81.5%)	31 (96.9%)	
- 1:Dead	12 (18.5%)	1 (3.1%)	
**Pathologic stage**			0.631
- stage i	12 (18.8%)	5 (15.6%)	
- stage ii	42 (65.6%)	21 (65.6%)	
- stage iii	7 (10.9%)	6 (18.8%)	
- stage iv	2 (3.1%)	0 (0.0%)	
- stage x	1 (1.6%)	0 (0.0%)	
**T stage**			0.725
- t1	15 (23.1%)	7 (21.9%)	
- t2	44 (67.7%)	21 (65.6%)	
- t3	4 (6.2%)	4 (12.5%)	
- t4	1 (1.5%)	0 (0.0%)	
- t5	1 (1.5%)	0 (0.0%)	
**N stage**			0.892
- n0	42 (64.6%)	19 (59.4%)	
- n1	16 (24.6%)	9 (28.1%)	
- n2	4 (6.2%)	3 (9.4%)	
- n3	3 (4.6%)	1 (3.1%)	
**M stage**			0.78
- mx	1 (1.5%)	0 (0.0%)	
- m0	62 (95.4%)	31 (96.9%)	
- m1	2 (3.1%)	1 (3.1%)	
**Race**			0.852
- asian	4 (6.5%)	1 (3.6%)	
- black or african american	8 (12.9%)	4 (14.3%)	
- white	50 (80.6%)	23 (82.1%)	
**ER status**			0.565
- negative	56 (87.5%)	25 (80.6%)	
- positive	8 (12.5%)	6 (19.4%)	
**PR status**			0.247
- indeterminate	1 (1.6%)	0 (0.0%)	
- negative	59 (92.2%)	26 (83.9%)	
- positive	4 (6.2%)	5 (16.1%)	
**HER2 immunohistochemistry level**			0.411
- 0	12 (31.6%)	5 (31.2%)	
- 1+	15 (39.5%)	7 (43.8%)	
- 2+	11 (28.9%)	3 (18.8%)	
- 3+	0 (0.0%)	1 (6.2%)	
**HER2 neuimmunohistochemistry receptor status**			0.82
- equivocal	10 (17.5%)	4 (17.4%)	
- indeterminate	1 (1.8%)	0 (0.0%)	
- negative	41 (71.9%)	18 (78.3%)	
- positive	5 (8.8%)	1 (4.3%)	
**Days**	1451.0 ± 1620.1	1850.7 ± 1875.0	0.281

SI Table S2. The thirty most significantly upregulated and downregulated DEGs with siControl vs. siCTGF

Upregulated genes			Downregulated genes		
Gene ID	Gen symbol	Fold change	Gene ID	Gen symbol	Fold change
3162	HMOX1	14.4525345	7498	XDH	0.031265893
101059953	NPIPA8	12.81924248	2838	GPR15	0.031613601
10912	GADD45G	10.65033954	79413	ZBED2	0.033886725
448831	FRG2	7.504160377	171425	CLYBL	0.036882476
2045	EPHA7	7.311635242	25984	KRT23	0.039749917
163479	FNDC7	6.850584216	25837	RAB26	0.04144623
2353	FOS	6.842931565	1591	CYP24A1	0.047173183
6520	SLC3A2	6.706840047	284086	NEK8	0.047465365
353355	ZNF233	6.572475417	282969	FUOM	0.052717378
4703	NEB	6.544985624	410	ARSA	0.063330653
285220	EPHA6	6.51685324	8343	H2BC7	0.063987892
135458	HUS1B	6.494670841	53405	CLIC5	0.0642736
2139	EYA2	6.430499325	3785	KCNQ2	0.067436025
56155	TEX14	6.423622603	9966	TNFSF15	0.068236291
83729	INHBE	6.134570455	2902	GRIN1	0.073049451
84992	PIGY	6.113645946	138429	PIP5KL1	0.079363042
66000	TMEM108	6.058969832	55349	CHDH	0.079494541
6615	SNAI1	5.994567395	51384	WNT16	0.079852867
93953	GCNA	5.731465109	259236	TMIE	0.080123197
57161	PELI2	5.697211517	167359	NIM1K	0.08037963
1960	EGR3	5.459356999	94059	LENG9	0.080554118
203102	ADAM32	5.327827562	4316	MMP7	0.080562454
100534592	URGCP-MRPS24	5.296230385	1003	CDH5	0.08192984
152206	CCDC13	5.26700851	653145	ANXA8	0.082626395
651	BMP3	5.209817309	5551	PRF1	0.082765997
202333	CMYA5	4.975310505	115908	CTHRC1	0.08325064
2495	FTH1	4.845459329	114800	CCDC85A	0.084696781
51655	RASD1	4.819380427	6573	SLC19A1	0.085245388
5630	PRPH	4.792693043	146429	SLC22A31	0.092053572
109504726	ERV3-1-ZNF117	4.763250988	2246	FGF1	0.097543352

Supplementary Figure S1

A

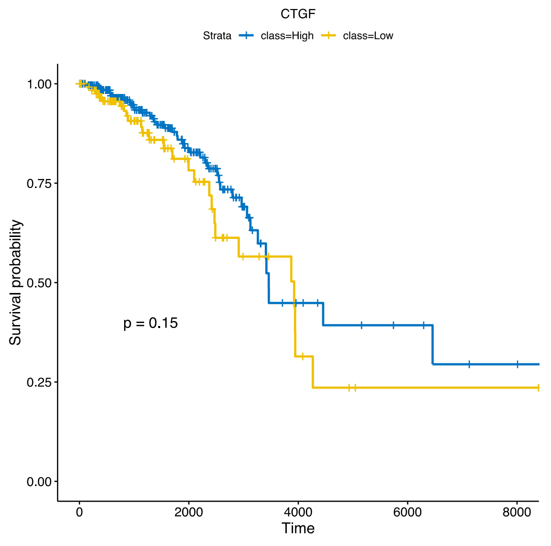

B

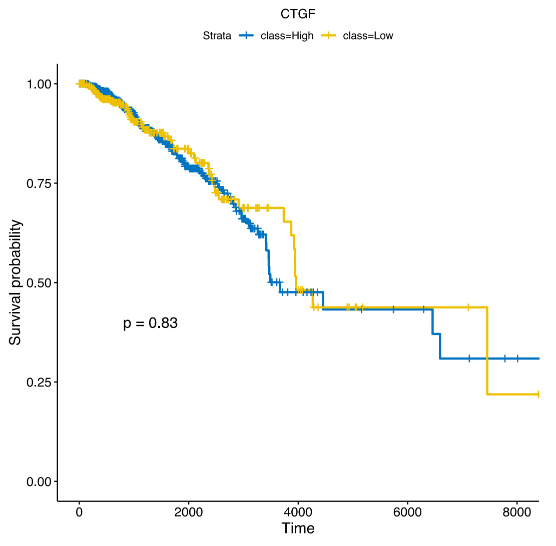

Figure S1. Kaplan Meier survival plots for BRCA and non-TNBC patient groups. (A) Kaplan-Meier survival plot for TCGA BRCA patient groups, CTGF mRNA high expression group (n=285) and low group (n=140). (B) Kaplan-Meier survival plot for the non-TNBC patient group, CTGF mRNA high expression group (n=731) and low group (n=360).

Supplementary Figure S2

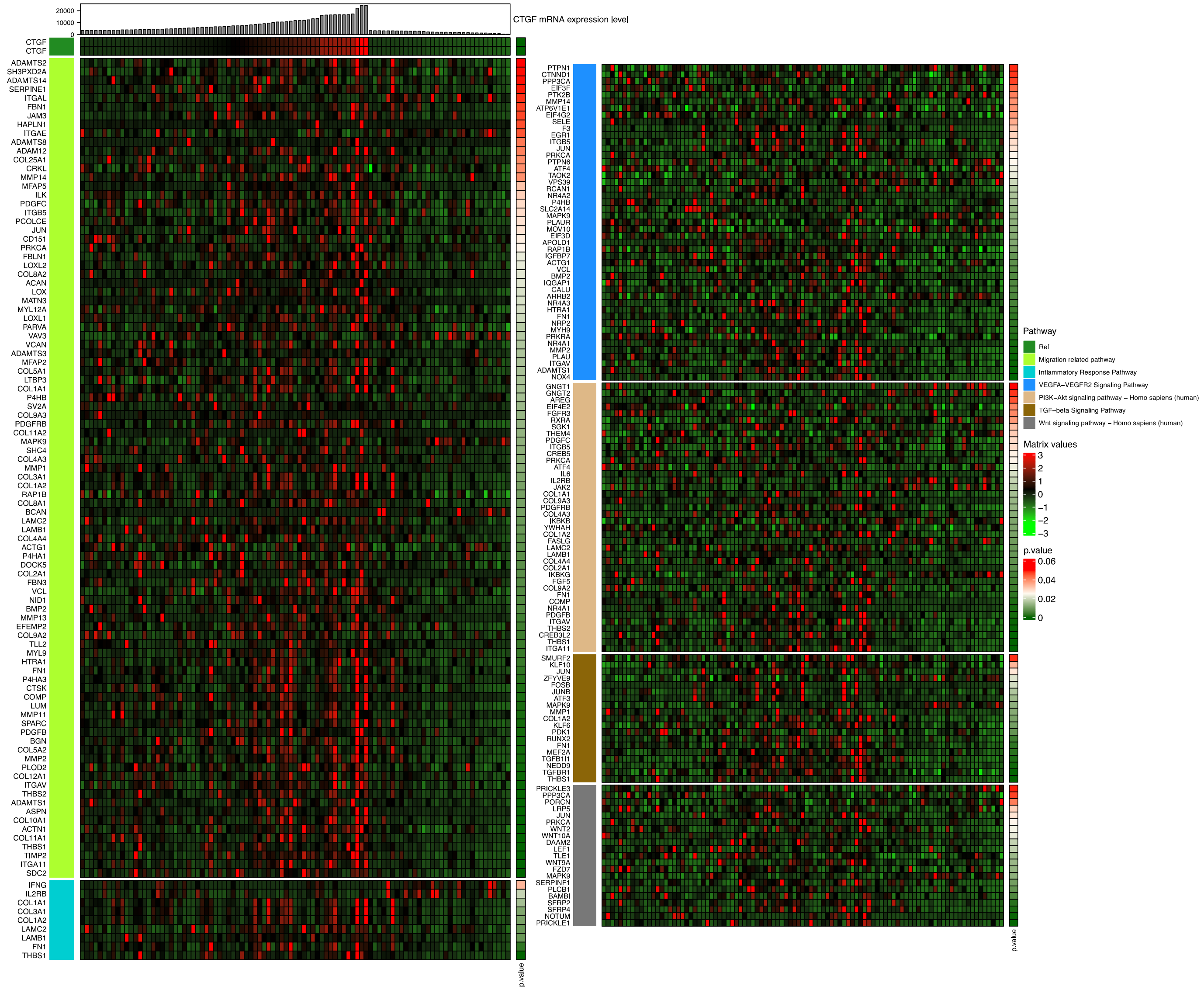

Figure S2. Over-representation analysis for CTGF associated pathways. Heatmap with selected pathways via CPDB (p-value<0.05 in over-representation analysis) based on mRNA expression in TNBC patients (n=97). Top annotation shows CTGF mRNA expression in TNBC patients (n=97). Rows represent the gene list for each selected pathway. Ten selected pathways are indicated next to the heatmap.

Supplementary Figure S3

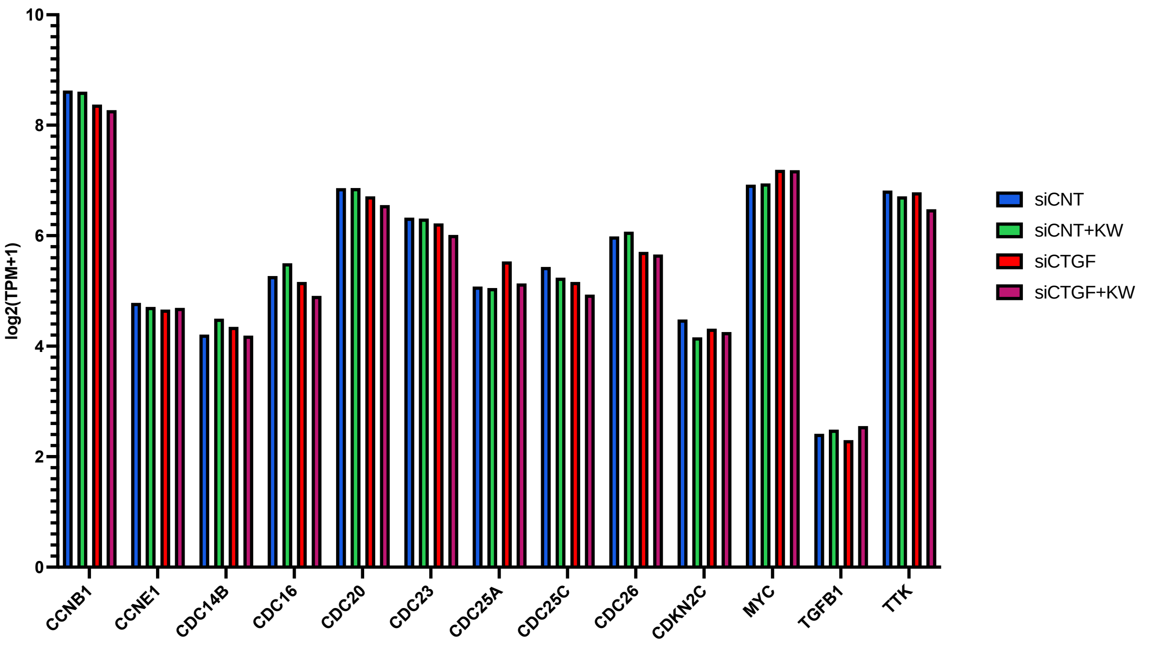

Figure S3. mRNA expression within cell cycle gene sets unaffected by kahweol. In the case of cells transfected with control siRNA, we identified 6174 genes (0.7 < FC < 1.3) through the comparison of kahweol treated cells and kahweol non-treated cells. Similarly, we selected 6229 genes (0.7 < FC < 1.3) compared between kahweol treated and kahweol non-treated in CTGF siRNA transfected cells. Over-representation analysis was performed by inputting overlapped genes between these gene sets that were unaffected by kahweol, and the prominent outcome pointed to gene sets related to the cell cycle (p-value = 0.002). The representative expression of genes in the cell cycle gene sets are visualized.

Supplementary Figure S4

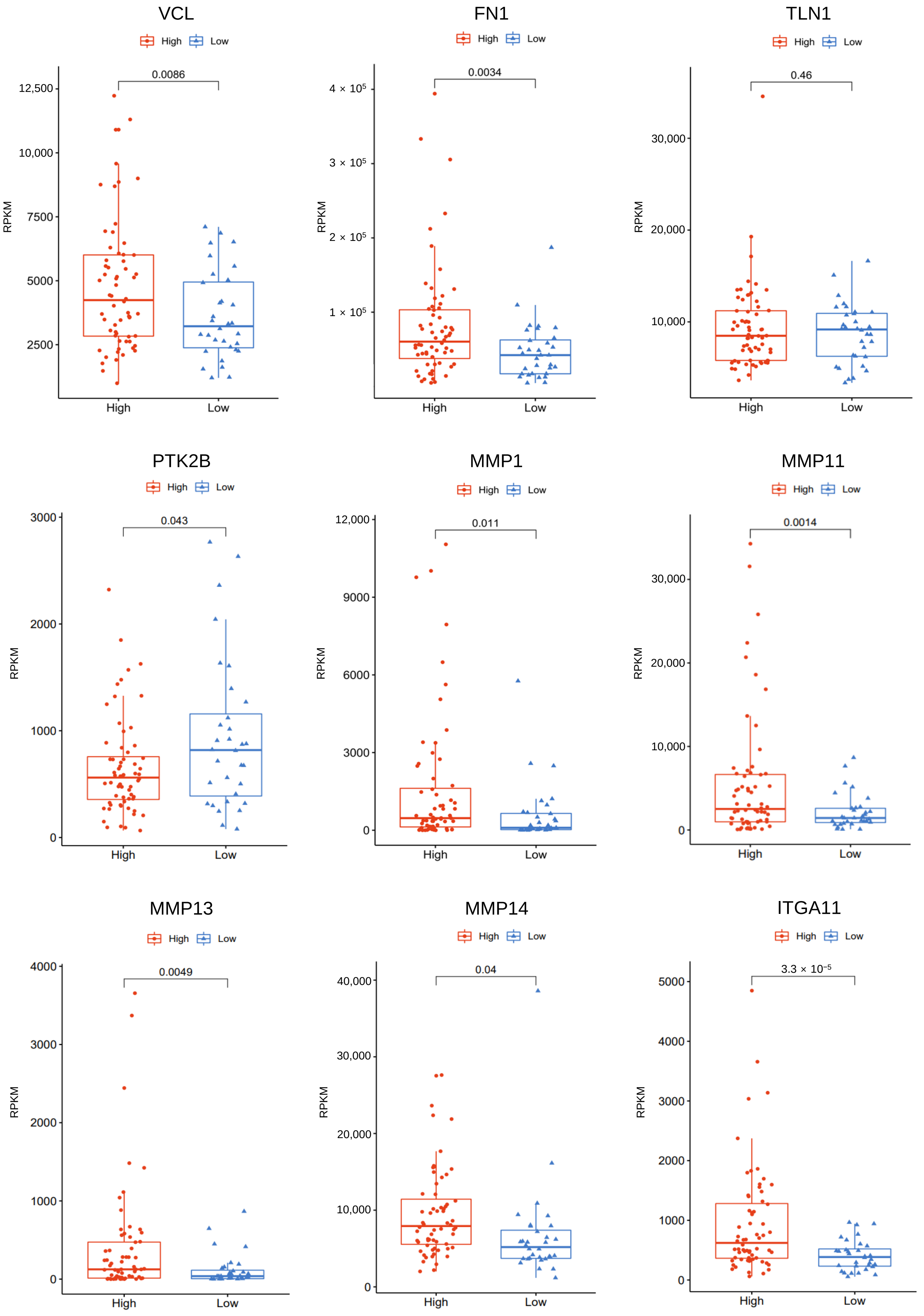

Supplementary Figure S4

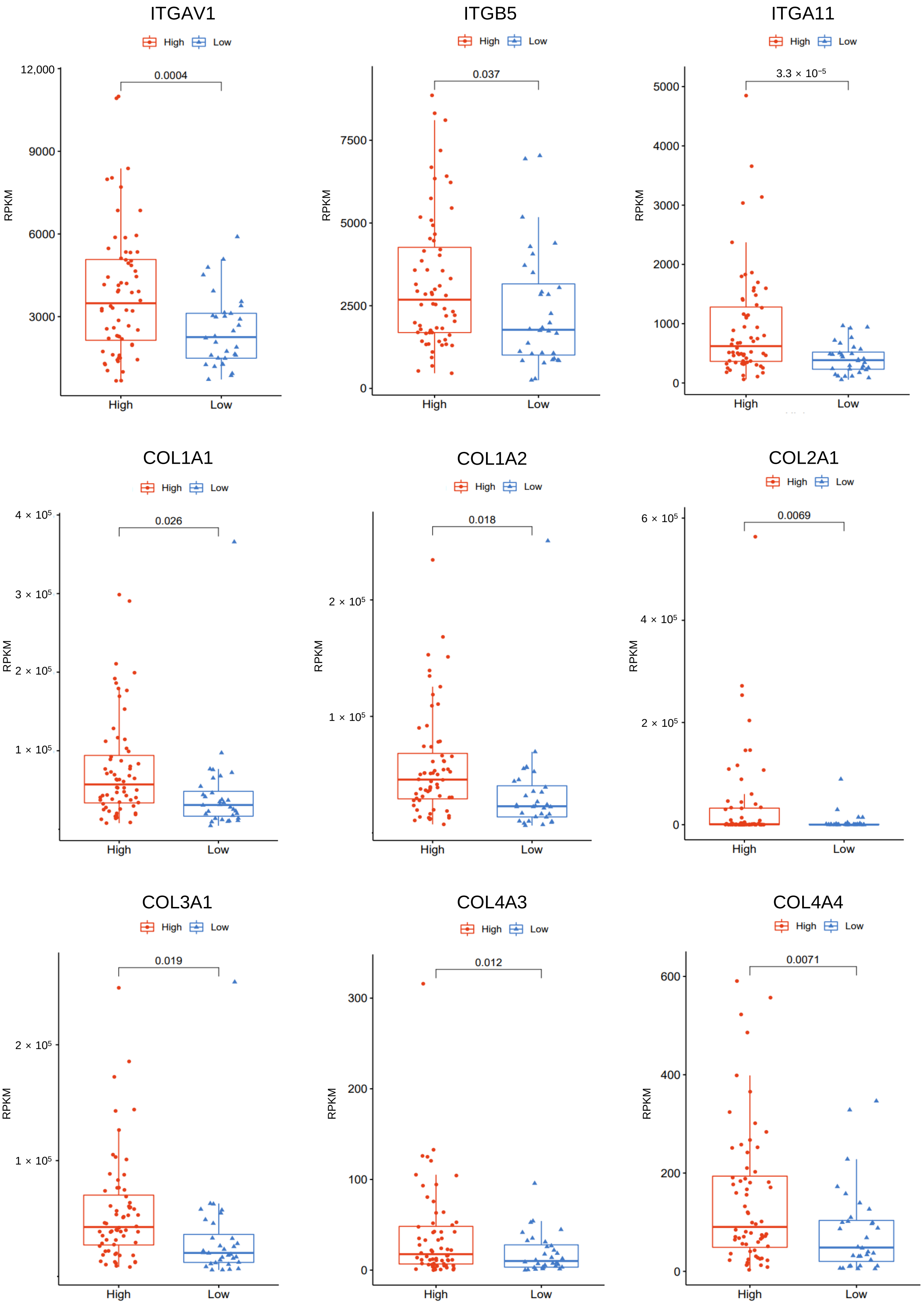

Supplementary Figure S4

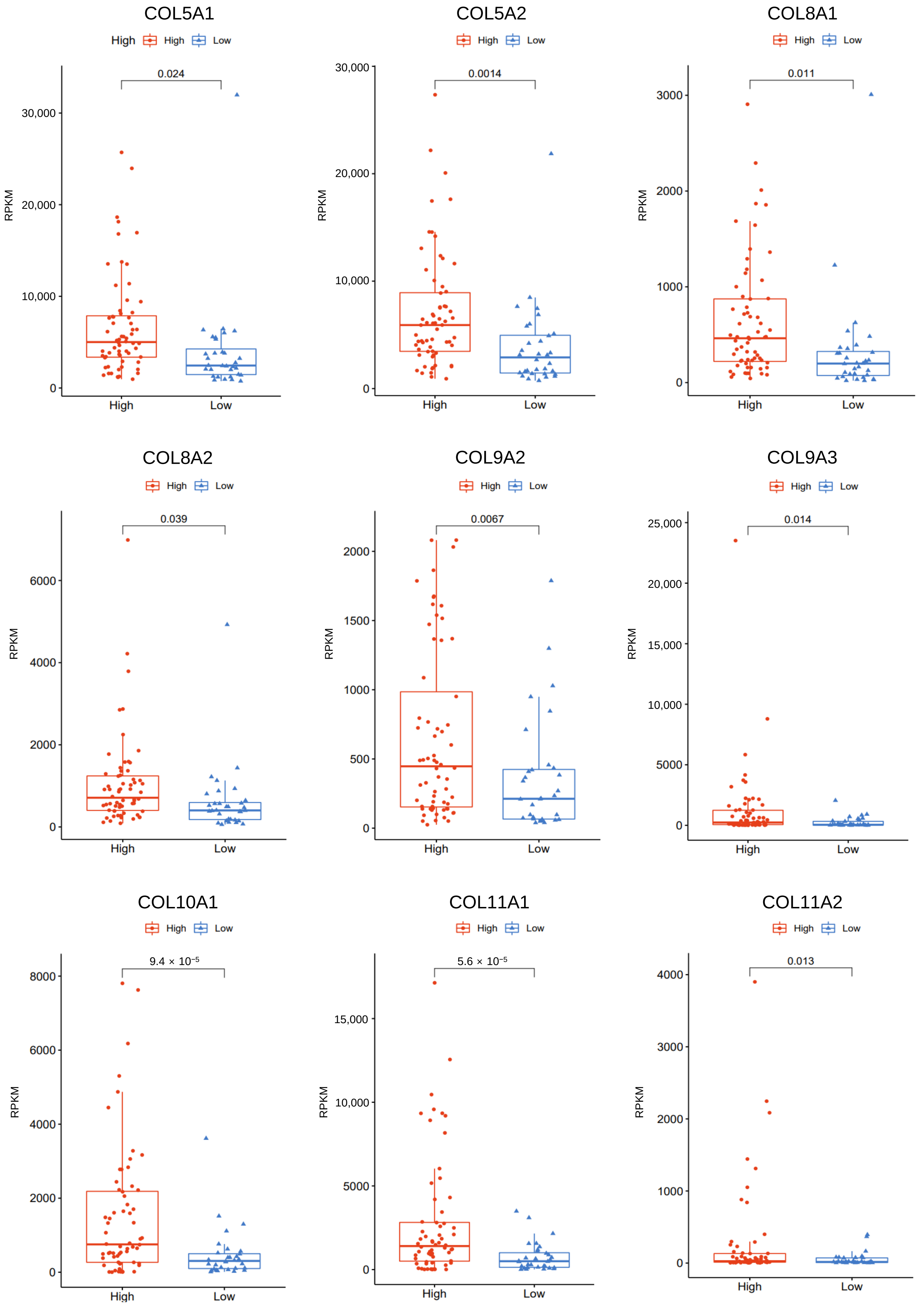

Supplementary Figure S4

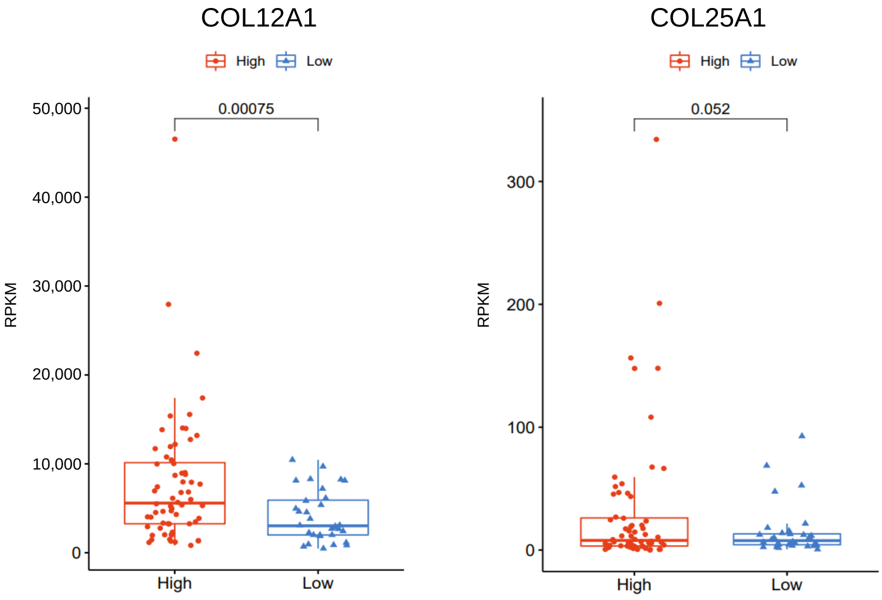

Figure S4. The expression of cell motility related genes in CTGF high and low group. Genes involving in migration related pathways based on transcriptomic analysis from TCGA BRCA patients.
